# Announcement

**DOI:** 10.1155/S1463924692000245

**Published:** 1992


### Announcement

### Major conference on analytical chemistry

To celebrate its 150th year, LGC is sponsoring, jointly with the Royal Society of Chemistry, a major international conference on analytical chemistry. The event, centred on major developments in analytical science and their impact, will take place at Reading University from 20–26 September 1992. The conference will be opened by Dr Richard Worswick, Government Chemist, whose speech will be entitled *The Laboratory of the Government Chemist: past and future.* A wide range of international specialists will be presenting the results of their work in more than 90 sessions throughout the week.

The event is based on a series of triennial conferences originally started by the Society for Analytical Chemistry (SAC). This year's programme will encompass papers and posters covering the whole field of analytical chemistry. Lectures will include *The development of an international measurement system,* by Dr Bernard King of LGC.

Special sessions will deal with laboratory quality assurance, accreditation and validation, and will be organized by LGC. The programme will include a visit by the international delegates to LGC. For a copy of a leaflet giving further information about the conference, contact **David Binney, LGC, 081 943 7423.**

